# Invasion potential of hornets (Hymenoptera: Vespidae: *Vespa* spp.)

**DOI:** 10.3389/finsc.2023.1145158

**Published:** 2023-05-09

**Authors:** Gard W. Otis, Benjamin A. Taylor, Heather R. Mattila

**Affiliations:** ^1^ School of Environmental Sciences, University of Guelph, Guelph, ON, Canada; ^2^ Institute of Bee Health, Vetsuisse Faculty, University of Bern and Agroscope, Bern, Switzerland; ^3^ Department of Entomology, Purdue University, West Lafayette, IN, United States; ^4^ Department of Biological Sciences, Wellesley College, Wellesley, MA, United States

**Keywords:** Asian hornet, extinction vortex, giant hornet, invasion potential, invasive species, propagule pressure, *Vespa*

## Abstract

Hornets are large, predatory wasps that have the potential to alter biotic communities and harm honey bee colonies once established in non-native locations. Mated, diapausing females (gynes) can easily be transported to new habitats, where their behavioral flexibility allows them to found colonies using local food and nest materials. Of the 22 species in the genus *Vespa*, five species are now naturalized far from their endemic populations and another four have been detected either in nature or during inspections at borders of other countries. By far the most likely pathway of long-distance dispersal is the transport of gynes in transoceanic shipments of goods. Thereafter, natural dispersal of gynes in spring and accidental local transport by humans cause shorter-range expansions and contribute to the invasion process. Propagule pressure of hornets is unquantified, although it is likely low but unrelenting. The success of introduced populations is limited by low propagule size and the consequences of genetic founder effects, including the extinction vortex linked to single-locus, complementary sex determination of most hymenopterans. Invasion success is enhanced by climatic similarity between source locality and introduction site, as well as genetic diversity conferred by polyandry in some species. These and other factors that may have influenced the successful establishment of invasive populations of *V. velutina*, *V. tropica*, *V. bicolor*, *V. orientalis*, and *V. crabro* are discussed. The highly publicized detections of *V. mandarinia* in North America and research into its status provide a real-time example of an unfolding hornet invasion.

## 1 Introduction

*Jacob Ishay, it seems, has started sending me Christmas presents from Israel. I assume it must be Ishay, for who else would have beautiful live queens of* Vespa orientalis *to give away? The strange thing is that he has packed them in crates of grapefruits and oranges and sent them via two British supermarkets!*

*I doubt if any exotic species will breed in Britain, for with our poor summer weather, I often wonder how anything breeds here! Still, it would be rather nice to find a colony of* Vespa mandarinia *in my back garden.*

Robin Edwards, Travelling Hornets (1)

Hornets in the genus *Vespa* (Hymenoptera: Vespidae) are conspicuous predators that form moderate to large annual colonies of eusocial insects. Several aspects of their biology facilitate their successful invasion of habitats in nonnative regions. In temperate regions, gynes initiate nest construction after undergoing a diapause period (2) during which they can be readily transported over long distances. They construct nests from widely available wood and plant fibers, exhibit little site specificity, and are non-territorial, a behavioral trait that could restrict population density (3, 4). Most species have cosmopolitan food preferences: adults consume sugary substances such as floral nectar and plant sap and capture diverse arthropod prey to feed their larvae (3, 5–7). Additionally, most have high reproductive rates (3). Plasticity of life history traits—such as colony size, colony longevity, polyandry, and polygyny—also facilitate their initial survival in new habitats (7, 8). Several species of *Vespa* have invaded regions outside their native ranges where they may disrupt natural communities by affecting local biodiversity and interfering with pollination systems, as has been demonstrated for several invasive species of social wasps in the genus *Vespula* (the yellowjackets) (9–11). Additionally, they may influence populations of other social wasps through competition and predation (12, 13). Several species attack honey bees during part or all of their colony cycle (14–22). *V. mandarinia* Smith, in particular, has been referred to as a top predator within its ecosystem due to its large size, exceptionally strong mandibles, and group hunting techniques (21).

All 22 species of hornets that comprise the genus *Vespa* occur in various regions of Asia (23). Only the ranges of two species, *V. crabro* Linnaeus and *V. orientalis* Linnaeus, naturally extend into Europe. No species are endemic to the Americas. However, in recent years, many hornet species have been detected in regions far from their endemic ranges. Of those, at least five species have successfully established non-native populations. The frequency of these incidents begs many questions: What are the most likely means of transport to new regions? How does the life cycle of hornets influence pathways of introduction? Does the invasion potential of hornets differ by species? And what biological factors have allowed some species to successfully colonize new regions while others have not?

In this article we summarize the records of *Vespa* species detected in exotic locations. We describe how certain aspects of their life history restrict introductions to a few stages of their annual colony cycle. We discuss a number of factors that contribute to successful establishment of exotic hornet populations in non-native locations. Finally, we synthesize these generalized patterns for the introduced species that have invaded new regions. Specifically, we consider how their biology and ecology (especially their overwintering strategy as gynes), propagule pressure and its genetic consequences, and the genetic diversity conferred to colonies by multiple mating by gynes likely contributed to their success. Throughout, we distinguish “queens”, reproductive females that have initiated nest construction or oviposition, from “gynes, females that have the potential to become queens.

## 2 Summary of exotic detections of *Vespa* hornets

Many hornet species have been reported from locations outside their native ranges (**Table 1**). In most instances where information about the discovery is available, individual hornets or single colonies have been detected, specifically: *V. affinis* (Linnaeus) in the United States (24) and New Zealand (25); *V. bicolor* Fabricius in United Arab Emirates (28); *V. crabro* in western Canada (29), Guatemala (30), and Mexico (31); *V. simillima* Smith in Canada (44) and Taiwan (45); *V. soror* du Buysson in Canada (29); and *V. orientalis* in multiple localities. Remarkably, the earliest record of a hornet transported long distance was a *V. bicolor* individual that survived the journey by boat from India to Marseille, France, in ~1800 (26).

**Table 1:** Location and year of detection of hornet (*Vespa*) species outside of their native ranges, excluding individuals and colonies found but destroyed during international border inspections.

Not established	Location	Year	Reference
*V. affinis*	United States	2010	(24)
	New Zealand	1997	(25)
*V. bicolor*	France	~1800	(26, cited by 27)
	United Arab Emirates	2022	(28)
*V. crabro*	Canada	2020	(29)
	Guatemala	<2010	(30)
	Mexico	2022	(31)
*V. mandarinia*	Canada	2019	(29)
	United States	2019	(32, 33)
*V. orientalis*	Brazil	1833	(34)
	Chile (established?^1^)	2018	(35)
	Czech Republic	<2017	(36)
	France	2021	(37)
	France (French Guiana)	1837	(34)
	Italy (north)	~2017	(38–41)
	Italy (Sardinia)	2022	(41)
	Madagascar	<1905	(34)
	Mexico	1998	(42)
	Romania	2019	(43)
*V. simillima*	Canada	1977	(44)
	Taiwan Island	2003	(45)
*V. soror*	Canada	2019	(29)
*V. velutina* ^2^	Yemen	<1997	(46)
Established			
*V. bicolor*	Taiwan Island	2003	(47, 48)
	Spain	2013	(27)
*V. crabro*	United States	~1840	(24, 49)
	Italy (Sardinia)	2010	(50)
*V. orientalis*	Spain	2018	(51, 52)
*V. tropica*	Micronesia (Guam)	2016	(53)
*V. velutina*	Western Europe	2004	(17, 54–56)
	Japan	2012	(57)
	South Korea	2003	(12, 58, 59)

^1^ The population of *V. orientalis* in Chile was first detected in 2018 (35). Numerous records distributed over ~1200 km^2^ (41) suggest that it may be established in the region surrounding Santiago.

^2^ Reported as *V. auraria* by Carpenter and Kojima (46).

Much less frequently, introductions of hornets develop into established populations in exotic locations. Once invaders are established, interregional accidental transport of gynes by vehicles and trains through what has been termed “jump-colonization”, along with flights by naturally dispersing gynes, combine to further expand the range of the species (e.g., *V. velutina* (60–63) and *V. orientalis* (38–41) in Europe). *V. velutina* is believed to have successfully established in Europe *via* the accidental introduction of a single gyne (58); the same may be true for its earliest colonization of Korea (64) and for *V. bicolor* in Taiwan (47). In comparison, information about the first steps of other hornet invasions, including analyses that determine the origin(s) of the original colonizers and genetic diversity within the invading population, are lacking.

Gynes of social wasps are detected infrequently in goods arriving at foreign ports of entry (9) and are destroyed before escaping into the surrounding environment. These border interceptions have not been included in **Table 1**. Smith-Pardo et al. (65) reported approximately two dozen interceptions of *Vespa* hornets, including *V. bellicosa* de Saussure, *V. orientalis*, *V. tropica* (Linnaeus), and a nest of *V. mandarinia*, during US border inspections between 2010-2018 (see also 29). In New South Wales, Australia, hornets of unknown species have been intercepted by quarantine border services (66). *V. orientalis* gynes have been detected in association with crates of fruit imported into Belgium and the UK from the Middle East (1, 67).

**Figure 1:**
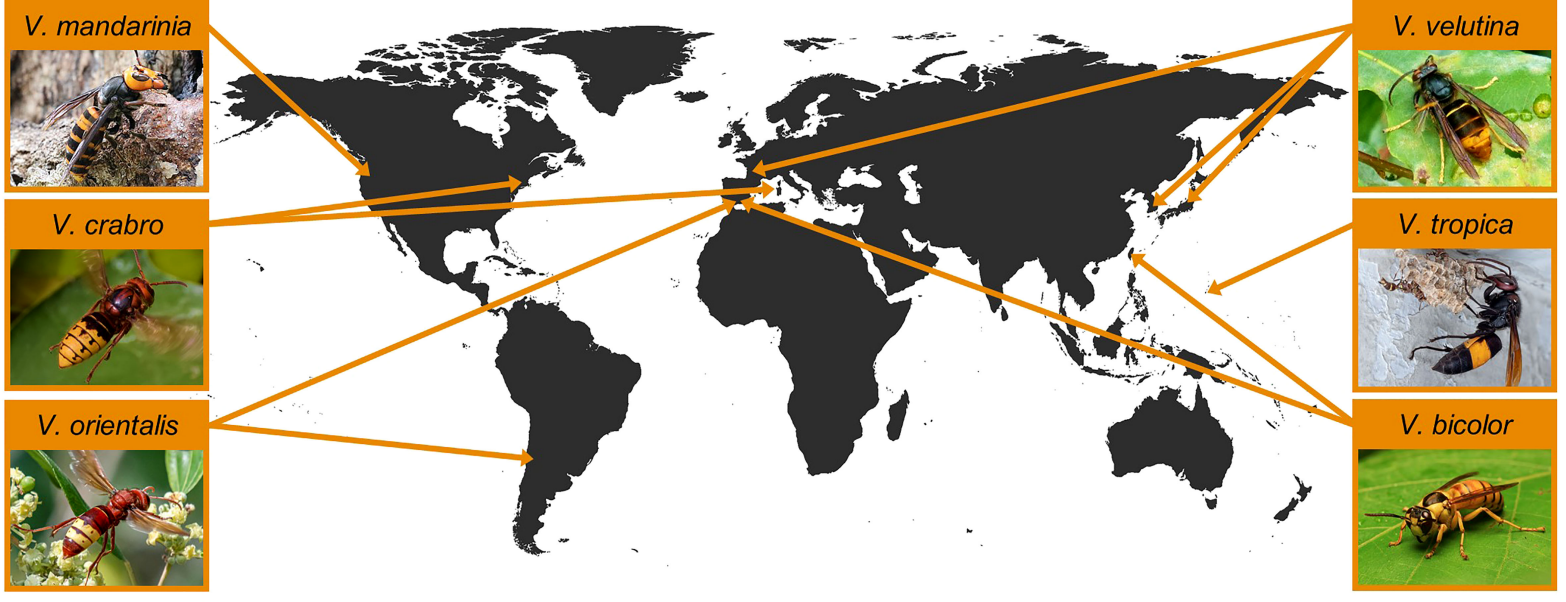
Sites of introduction of the five hornet (*Vespa*) species that have established non-native populations outside their region of endemicity and of the incipient population of *V. mandarinia*. Photographs, clockwise from the upper right, reproduced with permission from Patrick Le Mao, Vikrant Kumar, Kisa Wong, Adir Faduni Naduf, Ina Siebert, and Aline Horikawa, are available at the respective species accounts on iNaturalist.org.

Invasions of several species (**Figure 1**) deserve additional comments, on which we elaborate later in this review (Section 5). As mentioned, *V. velutina* has become invasive in western Europe and northeastern Asia following the accidental colonization of southwestern France and southeastern Korea by one or a small number of gynes approximately two decades ago. Subsequently, as those populations grew, local dispersal has resulted in numerous detections as the ranges expanded. The histories of these invasions have been summarized by several authors (e.g., 17, 57, 68) and we review them in section 5.1.

*Vespa orientalis* is notable for the many non-native locations in which hornets have been detected. These include several European countries, three widely separated countries in South America, Mexico, China, and Madagascar, as well as the United States during border inspections. More significantly, this species seems to have recently established populations in Chile (35, 41) and southern Spain (41, 51, 52, 69). These many detections and successful invasions suggest that the biology of *V. orientalis* must differ in a way that generates a high invasion threat in comparison to other *Vespa* species (discussed in section 5.2).

The giant hornets, *V. mandarinia* and *V. soror*, received exceptional press coverage and intensive search efforts following their discovery in western North America (**Figure 1**). In spring of 2019, a gyne of *V. soror* was collected at the harbor of Vancouver, British Columbia; this is the only detection of that species outside of its endemic range in subtropical Asia (29). That single hornet was a prelude to the media frenzy sparked by the discovery of both individual workers and active nests of its sister species, *V. mandarinia*, in British Columbia and Washington state between 2019–2021 (33). We discuss details of the incipient population of *V. mandarinia* in section 6.

## 3 Pathways of introduction of nonnative hornets

### 3.1 Accidental transport of diapausing gynes

The typical process of overwintering by gynes of *Vespa* and other vespid wasps facilitates their accidental long-distance transport as stowaways (9). The annual life cycles of all *Vespa* species are relatively similar (2, 21, 23, 70, 71). Towards the end of the colony cycle (i.e., in fall in temperate climates), colonies enter their reproductive period and produce gynes and males. After emerging as adults, those reproductives remain in the nest for a week or more, during which time they are fed by both adult workers and larvae. This feeding period is critical for the accumulation of large fat reserves required for the winter survival of diapausing gynes (21, 23). After leaving their nest and mating, gynes of most species individually search for overwintering sites, where they construct hibernacula most commonly in loose soil or rotting wood (21, 71). Data on hibernacula come predominantly from Matsuura (21, 71) who located most of the hibernacula of *V. mandarinia*, *V. crabro*, and *V. tropica* gynes in soil, and a majority of those of *V. simillima* and *V. analis* in rotting wood. A few wintering *V. mandarinia* gynes were located in heaped straw and one was found in an abandoned *V. crabro* nest (21, 72). However, in a few species several gynes may overwinter together in a single hibernaculum [e.g. two gynes in *V. analis* (71); up to ten gynes in *V. crabro* (73); groups of gynes in *V. orientalis* in cavities (16)]. Young gynes are believed to limit their dispersal distance in the fall prior to diapause (21), perhaps to conserve fat reserves. However there are few supporting observations to confirm that.

Studies of hornets in tropical regions are limited. Several reports indicate that annual cycles can be asynchronous, with colonies in all stages of development occurring in both the dry and rainy seasons (74, 75). It is unknown whether gynes enter diapause or initiate nesting activities soon after eclosing and mating (75).

While in pre-nesting diapause, mated gynes are inactive and subsist on internal reserves of lipids acquired before departing their natal nest and stored in their fat body (2, 76). The amount of fat a gyne has stored affects her survival during diapause and may influence nest founding (3, 77). During this period, which can vary from four to eight months depending on the species and climate of their native environment, quiescent gynes have the potential to be transported accidentally over long distances in shipments of commercial goods, making this the most common means of transoceanic dispersal in *Vespa* and other social wasps (9). Most of the species in **Table 1** likely colonized overseas regions when stowaway gynes became active after being shipped during their diapause. In the best-known example, the population of *V. velutina* that now inhabits most of western Europe is believed to have been founded when a single, multiply mated gyne was shipped to southwestern France in pottery imported from China, in or before 2004 (58, 78). The *V. simillima* gyne collected on the west coast of Canada likely arrived with wood imported from Japan (44). Wood shipments are also the suspected pathway by which *V. crabro* arrived in Sardinia from mainland Italy (50). Similarly, a *V. velutina* wasp was found dead in a load of wood that had been treated with boron prior to shipment from France to the UK (79). *V. orientalis* gynes caught inside grocery stores in Belgium and England were associated with shipments of citrus fruits from the Middle East; details about how these gynes arrived with fruits are not available (1, 67). Matsuura’s observation that gynes can build hibernacula in heaped straw (21, 72) invokes the possibility of gynes doing the same in other types of loose packing material.

Increasing volumes of maritime trade over the last 50 years are positively correlated with increases in the number of introductions of non-native species (80–84). In the case of hornets, dormant gynes in cavities, between logs and under bark, in soil associated with nursery stock, and less frequently in straw and hay would likely escape detection during inspection at ports of entry (85 and references therein). Moreover, because dormant gynes of most species create hibernacula underground or in protected sites, they may escape treatments with insecticides applied to kill unwanted plant pests.

Because air freight may be stored in unheated conditions and the cargo hold of planes is generally kept cool, diapausing gynes might remain quiescent during the transport of air freight. This scenario may have occurred in the case of a *V. velutina* individual that was detected in an air cargo area at the Paris airport and subsequently destroyed (86). Other diapausing gynes may have escaped detection and become successful invaders.

### 3.2 Dispersal by active gynes at the start of the annual colony cycle

Following diapause (i.e., in spring in north temperate regions), gynes enter a pre-nesting period of feeding and dispersal that may last several weeks before they initiate nest construction (21). With their fat deposits largely depleted, they fuel their metabolism almost exclusively with carbohydrates (76) foraged from tree sap and floral nectar (21). Matsuura marked hundreds of hornet gynes over multiple years at sources of tree sap, but within a few days of marking he failed to recapture any of them, suggesting they had dispersed some unknown distance away (21). Hornets are strong fliers with good dispersal ability (3). Studies of the range expansion of the population of introduced *V. velutina* in Europe provide rather variable estimates of natural dispersal distances during the pre-nesting period. These range from ~78 km/year in France (in the absence of significant geographical barriers) (62), to averages of 20, 37, and 45 km/year in Portugal (depending on the direction of spread) (63, 87). Although gynes dispersed at a rate of 18 km/year along the northwestern coast of Italy (60), it was estimated that very few of them dispersed more than 12 km without human assistance (88). Beggs et al. (9) commented that gynes in spring may be able to fly ~30 km/day. In agreement with that estimate, gynes of *V. velutina* are believed to have flown have flown across the sea from France and colonized under their own flight power several of the Channel Islands located 15–40 km from the mainland (61). Along with natural dispersal by gynes, once a population has become established, human-mediated “jump dispersal” *via* unregulated transport of goods by trains, trucks, and cars can greatly enhance the rate at which its range increases (62, 63, 89). In Korea, *V. velutina* has expanded its range at the rate of 10–20 km/year (12, 90). We are not aware of similar estimates for natural dispersal distances for any other hornet species.

### 3.3 Accidental introduction of entire colonies

Live colonies of hornets are unlikely to be accidentally transported by humans. First, hornets do not store food in their nests (91). Rather, their larvae convert proteins from animal prey into amino acid-rich sugars, so they serve as living reservoirs of food for adult hornets during dearth periods (21, 23, 92). If adult wasps were unable to forage during transit, larvae could continue to supply sugars to adults for only a few days, after which time the colony would perish. Moreover, established *Vespa* colonies metabolize carbohydrates to regulate their internal nest temperature (71, 93). Colony thermoregulation would further deplete food reserves and make it even more likely that hornet colonies would starve during transport. Secondly, an active nest of hornets with numerous large, defensive insects is likely to be discovered and destroyed, either while in transit or by inspectors upon arrival in a port. Although accidental transport of live colonies may be theoretically possible, it is an infrequent pathway of introduction relative to stowaway diapausing gynes.

### 3.4 Introduction of gynes *via* shipments of fruits and vegetables

Shipments of fruits and vegetables represent a negligible pathway of entry because hornets are not expected to remain with fruit during cleaning and packaging (94). Nevertheless, live *V. orientalis* gynes detected many years ago in grocery stores in Belgium and the UK were associated with shipments of oranges and grapefruits from the Middle East (1, 67). They likely were in diapause when transported as they were found in the months of December and January. This confirms that the low risk of importation of some species in association with imported produce can be realized.

### 3.5 Human importation of hornet brood

The brood of *Vespa* hornets is considered a culinary delicacy in many parts of Asia (95–98). Comb *containing V. mandarinia* larvae and pupae that was intercepted at a US port of entry (65) was probably being imported as food. USDA-APHIS concluded that “hornet comb containing pupae for consumption is a likely path for introduction” of *V. mandarinia* (99). Contrary to that assessment, constraints imposed by the biology of hornets make it highly improbable that imported larvae and pupae, in the absence of adult hornets, could give rise to mated gynes capable of surviving winter diapause.

In the absence of caregiving workers and nutrient-rich larval hornets, isolated gynes and males that may survive and emerge from pupae in imported comb would not be able to acquire the amino acids and store the fat they require to thrive. During their diapause, gynes survive primarily by metabolizing extensive reserves of stored fat [e.g., *V. orientalis* (76)]. In nature, young gynes and males remain in their nest for one to two weeks before they leave to mate (21). During this period, they receive large quantities of food from both workers and larvae (21, 76) and their fat bodies become noticeably enlarged in both temperate and tropical *Vespa* species (summarized in 23; see also 76). In the case of gynes, their mass increases dramatically—by 20–40%—mostly due to the deposition of fat (21, 23). For example, *V. mandarina* gynes increase in mass from 2.88 ± 0.25 g to 3.46 ± 0.21 g after emerging as adults (2). Gynes of *V. affinis* increase in mass by 40%, from 0.64 ± 0.08 g to 0.90 ± 0.02 g over a period of two weeks after eclosion (100). Most of this fat is metabolized during diapause; loss of mass by diapausing gynes ranges from 30–40% in the species that have been studied (21, 23, 76). In *Vespula vulgaris* (Linnaeus), the quantity of fat stored by gynes affects their subsequent quality as queens and their likelihood of successful nest establishment (101). Male hornets, because they are incapable of digesting protein, rely on amino acids received in oral secretions from larvae for their maturation into sexual adults (102).

## 4 Factors influencing establishment of adventive populations of hornets

### 4.1 Propagule pressure

Propagule pressure at a location is a function of the number of reproducing individuals of a species introduced (“propagule size”) and the number of times the species is introduced (“propagule number”) (103–105). It has a large impact on whether an introduced species becomes successfully established (3, 104–106). For example, in a meta-analysis of approximately 1,000 publications related to invasive species, propagule pressure was the factor most strongly correlated with their establishment (107). It is well recognized that propagule size influences successful establishment of insects that are intentionally introduced as biological agents (e.g., 108, 109). In the case of hornets, a single fertilized gyne is unlikely to succeed in establishing a new population (105) except for a few species under exceptional circumstances, as likely occurred with *V. velutina* in Europe (9, 58). Unfortunately, the propagule pressure of most exotic species, including that of hornets, is essentially unknown because of the low probability of detection (85, 110–112), the biased nature of their interceptions due to inspections being primarily directed towards quarantine pests (61), and the failure of inspection agencies to make the data on their detection at international borders accessible. In the case of most species of hornets, the numbers of gynes transported to any given locality within a period of a few years is probably low. This low propagule pressure explains in part why relatively few populations of hornets have become established, despite the relative ease with which gynes can be transported over long distances while diapausing (reviewed above) (9). An exception may be *V. orientalis*, in which gynes often overwinter in groups (16). That behavior would result in larger propagule size and correspondingly greater genetic diversity in founding populations. Increased quantities of goods shipped internationally have undoubtedly increased the propagule pressure of hornets worldwide.

Directly related to propagule pressure is the abundance of a species in its endemic range (103), which in the case of hornets affects the likelihood of gynes arriving accidentally at exotic locations. Most of the hornet species that have been detected outside their region of endemicity and all five established invaders (**Table 1**) are relatively common inhabitants at low elevations, and often reside in human-disturbed habitats within their native ranges. In contrast, uncommon and rare species (e.g. *V. basalis* Smith*, V. bellicosa*, *V. fervida* Smith, *V. fumida* van der Vecht, *V. multimaculata* Pérez, *V. philippensis* de Saussure), species inhabiting high elevation forests (e.g. *V. binghami* du Buysson, *V. luctuosa* de Saussure, *V. mocsaryana* du Buysson, *V. vivax* Smith) (65, 113), species with small colonies that produce few gynes (e.g. *V. ducalis* Smith) (14), and the uncommon social parasite *V. dybowskii* André should be less frequently transported accidentally. Of all these species, only *V. bellicosa* has been intercepted outside its endemic range (65). It is an uncommon inhabitant of tropical lowland rain forests of Borneo and Sumatra (114) and, as such, is an unlikely species to be transported transoceanically. Its detection at the border of the US indicates that most if not all *Vespa* species have the potential to be moved long distances by humans, especially with high and increasing volumes of international trade.

### 4.2 Genetic diversity of introduced hornet populations

Invasion genetics is a large and expanding field (reviewed by 115) and close attention to how genetics influences non-native populations of hornets is needed to understand their invasion potential. Colonists are unlikely to find themselves in an environment identical to that from which they originated. Generalist predators like social wasps will exhibit some phenotypic plasticity in behavioral and life history traits that may enhance their success in a novel environment (7, 116, 117). Moreover, following colonization, mathematical modeling predicts that they may experience a rapid increase in phenotypic plasticity (118). However, genetic adaptation to the conditions in a new locality is also important to invasion success in most instances (119). Additive genetic variance provides most of the raw material that allows an incipient population to respond to natural selection in the newly colonized location (120); epistatic variation may also play a significant role (119). Of particular importance are loci that have large phenotypic effects or cryptic variation (i.e., genes that were not under selection in the source environment) that is exposed following introduction (121). Founder effects and genetic bottlenecks at the site of introduction that result from small numbers of colonists reduce genetic diversity and may negatively affect the success of incipient populations (120, 122). Such populations will consequently experience genetic drift, stochastic loss of allelic diversity, and Allee effects (123) in proportion to propagule size and the rate of population increase of the introduced species in the new habitat (124). These factors are difficult to assess for *Vespa* hornets because, aside from *V. velutina* in Europe (79, 125), the genetic diversity of exotic hornet populations has not been analyzed.

Additionally, mating of related individuals within small populations generally results in inbreeding depression and often leads to local extinction (i.e., failure to establish) (106, 121, 126, 127). Mating by *Vespa* queens with one male, which is the norm in several species (**Table 2**), further limits the amount of genetic variability carried by gynes and subsequently present within colonizing colonies. If only a few, widely distributed founding gynes colonize a region, there will be a high probability of brother-sister matings in the subsequent generation. Extreme inbreeding has negative consequences within the Hymenoptera (discussed in section 4.3). In contrast, polyandry (when a gyne mates with multiple males) is common in *V. velutina* (58, 139), *V. simillima* (129, 130), and possibly other species in the *bicolor* phylogenetic group (141), such as *V. bicolor.* Polyandry increases within-colony genetic diversity logarithmically with increasing number of mates (142). In other eusocial insects, polyandry has been shown to increase colony fitness and productivity (143, 144), colony growth rate (144, 145), resistance to infection by disease (146, 147), and the capacity of colonies to respond to environmental changes (148). Polyandry is predicted to reduce the probability of a population experiencing a diploid-male extinction vortex (discussed in section 4.3) (149, 150). Simultaneous introduction of several gynes that are wintering together (e.g., as occurs fairly often in *V. orientalis*; 16) would also augment genetic variation in incipient populations.

**Table 2:** Summary of mating frequency by gynes of nine species of hornets (*Vespa* spp.).

Species	Location	Mean Mating Frequency^1^ ± SD (n)	Number of Mates (Range)	Mean Effective Paternity^2^	Reference
*V. affinis*	Japan (Iriomote)	1.50 ± 0.76 (14)	1–3	1.3 ± 0.45	(128)
	Japan (Iriomote)	1.1 (20)	1–2	–	(129)
*V. analis*	Japan (Hokkaido)	1.0 ± 0 (20)	1	1.0 ± 0	(130)
	Japan (Honshu)	1.10 ± 0.09 (20)	1–2	1.05 ± 0.03	(131)
*V. crabro*	United Kingdom	1.43 ± 0.65 (14)	1–3	1.11	(132)
	United Kingdom	1.14 ± 0.36 (21)	1–2	1.04	(133)
	Japan (Honshu)	1.35 ± 0.67 (20)	1–3	1.13	(134)
	Japan (Hokkaido)	1.1 (14)	1–4	–	(129)
	Germany^3^	1.31 ± 0.63 (13)	–	–	(135)
*V. ducalis*	Japan (Honshu)	1.0 ± 0 (20)	1	1.0	(136)
*V. dybowskii*	Japan (Honshu)	1.13 ± 0.52 (15)	1, 3	–	(129)
*V. mandarinia*	Japan (Honshu)	1.1 ± 0.09 (20)	1–2	1.03 ± 0.02	(137)
*V. simillima*	Japan (Honshu)	2.14^4^	1–4	2.38^4^	(128, 129)
	Japan (Hokkaido)	1.53 ± 0.74 (15)	1–3	1.33 ± 0.74	(130)
*V. soror*	Vietnam	1.0 ± 0 (4)	1	1.0 ± 0	(138)
*V. velutina*	France	2.44–4.11 (9)^5^	1–5 or more^5^	—	(58)
	United Kingdom	1.57 ± 0.79 (7)		—	(139)

^1^ Mating frequency is the arithmetic mean number of matings per gyne. Absence of a standard deviation indicates the value was omitted in the original source or it could not be calculated because the raw data were not presented.

^2^ Mean effective paternity is the harmonic mean of the number of matings per gyne (140).

^3^ Hoffmann et al. (135) observed matings between 13 female and 10 male *Vespa crabro* for 2 hours per day over 6 days, in a 50 x 50 x 50 cm screen cage.

^4^ Takahashi (129) reported the arithmetic mean mating frequency. Martin et al. (128) cited Takahashi (129) as the source of data from which effective paternity was calculated.

^5^ Arca et al. (58) reported estimated ranges of mating frequency per queen. One queen was estimated to have mated 5–8 times. All matings occurred in nature, with the exception of the data for *V. crabro* in Germany. If authors included their data on mating frequency, the arithmetic mean and standard deviation were calculated.

The number of different geographic sources contributing to an incipient population of hornets will also affect the trajectory of that population. The increased genetic diversity that results from admixture from different source populations will provide novel genotypes upon which natural selection can act. However, differing outcomes are possible. In many if not most cases, admixture will increase the potential of an exotic population to adapt to its new environment (119, 121, 151) and potentially to expand its range rapidly (152). On the other hand, incompatibilities between individuals derived from genetically disparate source populations may result in low fitness of intraspecific crosses (119, 121). To date, the influences of different geographic sources and the fitness of admixed populations have not been studied in hornets.

### 4.3 Complementary sex determination and associated diploid-male extinction vortex

All Hymenoptera, including hornets, have haplodiploid sex determination, in which males arise from unfertilized (haploid) eggs and females (workers and queens) develop from fertilized diploid eggs (153). In most haplodiploid species, the actual sex-determination system involves one gene (“single locus”) at which there are usually many different complementary sex-determining alleles (variants of the gene) within a population (154). For an egg to develop into a female, it must not only be fertilized, it must also be heterozygous at the sex-determining locus (i.e., the alleles from the queen and male must differ). This system is known as “single-locus complementary sex determination” (i.e., sl-CSD) (154).

Gynes of most hornet species usually mate with just one male (**Table 2**), and for them, sl-CSD presents yet another obstacle to successful colonization. When a gyne mates with a single male and his single sex-determining allele is the same as one of the two she bears, then approximately 50% of the fertilized eggs she lays, if reared to adults, will develop into diploid males rather than females. These diploid males impose a genetic load on the colony because their production utilizes energy and resources, but they perform no functional tasks in their colony (155, 156). During the reproductive phase of the colony, these diploid males may mate with gynes. However, because they are sterile or have low viability, they sire few triploid, sterile daughters, and therefore impose an additional genetic load on the population (157, 158). Diploid males are commonly encountered in the invasive population of *V. velutina* in Europe (79, 139, 155), indicating that the population has a small number of sex-determining alleles. Additionally, their production suggests that hornets in general have sl-CSD and that diploid male production is very likely to occur in situations with extreme inbreeding for other hornet species as well. Despite the constraints imposed on hymenopteran invasions by sl-CSD, the invasive success of *V. velutina* in Europe demonstrates that they can be overcome if other elements influencing the colonization are favorable.

The sl-CSD system results in an Allee effect (i.e., a decrease in mean individual fitness with decreasing population size) that is predicted to increase the vulnerability of small populations to extinction (159). The genetic load caused by the production of diploid drones places those colonies at a selective disadvantage that may reduce the size of an incipient invasive population. The population is predicted to enter a negative feedback loop, with progressively smaller population sizes leading to further stochastic reductions in diversity of sex-determining alleles, in what has been termed a “diploid male extinction vortex” (DMV) (156, 158). In practice, DMVs are unlikely to be the sole cause of extinction of incipient populations of hornets; however, they threaten population persistence when combined with the other effects caused by extreme genetic bottlenecks under specific ecological circumstances (150). As some hornet introductions have not resulted in the establishment of successful populations and many have likely gone undetected, the contribution of DMVs to their extinction cannot be estimated (160).

Two conditions serve to increase genetic variability and potentially elevate the invasion potential of hornets. First, hymenopteran populations founded by multiply mated (polyandrous) queens have substantially greater probabilities of establishment due to increased overall genetic variability and diversity of sex-determining alleles in their colonies (3, 161, 162). The success of *V. velutina* in Europe has been partially attributed to polyandrous mating by the initial foundress queen with several (probably four) males (58). Even in hornet species for which the large majority of gynes are singly mated, such as *V. mandarinia* and *V. crabro*, a minority of individuals exhibit polyandry by mating with two or three males (**Table 2** and references therein). Secondly, multiple introductions of gynes from genetically distinct populations result in greater genetic diversity in adventive populations (151). For example, the *V. mandarinia* individuals that recently colonized North America originated from different regions of Asia (163). Had these introduced hornets been located in close proximity to one another and interbred, their offspring would have been genetically admixed, which might have influenced the trajectory of that incipient population. In Korea, genetic analyses of invasive *V. velutina* indicate that a small number of independent colonization events occurred in different regions of the country (164), which may have contributed to the species becoming well established there and in southeastern Japan. Research to determine the number of different queens that have been introduced to a site and the number of source populations, as well as additional traits such as levels of heterozygosity, numbers of matings by gynes, the number of sex-determining alleles, and confirmation of the number of CSD loci, are needed to better understand the effects of these genetically influenced factors on hornet invasions. At present, data for most of these variables are available for one species only, *V. velutina* in Europe and Korea+Japan, where the invasive populations may be evolving rapidly (58, 139).

### 4.4 Natural enemies: parasites, parasitoids and pathogens

Parasites, parasitoids, and pathogens have the potential to reduce the growth rates of hornet infestations. Because low genetic diversity following population bottlenecks reduces resistance to parasites and pathogens, invading hornets should in theory be particularly vulnerable to such stressors (165, 166). However, solitary gynes may also gain an advantage because they are likely to leave behind some parasites and predators during long distance colonization events (3). Furthermore, invading hornets, if already infected by pathogens, may facilitate and increase the negative impacts of their invasions by acting as “biological weapons” if they arrive infected by pathogens that affect native vespid populations more adversely, thereby reducing their competitive interactions (167, 168). For vespids, possible interactions with parasites and diseases—both natural and newly encountered species—are numerous. Turchi and Derijard (169) reviewed the potential for natural enemies (strepsipterans, fly parasitoids, nematodes, mites) and pathogens (i.e., *Beauvaria bassiana*) to control *V. velutina*. Matsuura and Yamane (21) presented an extensive list of the parasites and parasitoids that infest hornets in Japan, noting that their effects on colonies are not particularly harmful or pervasive. Additionally, numerous viruses have been detected in social hornets (170–174). Marzoli et al. (171) commented on the apparently high efficiency of viral transfer from bees to hornets, in many cases apparently through consumption of infected bees by hornets. Despite these seemingly common viral infections, there are very few reports of overt symptoms of disease in infected hornets (e.g., Deformed Wing Virus in a *V. crabro* gyne; 175). The effects of viruses on hornets and their colonies are currently unknown.

To summarize for hornets in general, low prevalence and abundance of parasites and parasitoids coupled with little evidence of pathogenesis in hornets suggest that natural enemies are unlikely to control populations of invasive hornets except perhaps in exceptional situations.

### 4.5 Human destruction of gynes and colonies

Trapping mated gynes in spring theoretically has the potential to reduce populations of hornets. Unfortunately, in practice, all attempts to control established populations of social wasps that were focused only on trapping gynes have proven inadequate [e.g., *Vespula germanica* (Fabricius) in New Zealand, 176); *Polistes chinensis* (Fabricius) in New Zealand, 177); *Vespa velutina* in Europe, 178–181)]. Hornet traps generally consist of a container, baited with fresh protein (e.g., sardines, finely ground beef) or an attractive scented liquid (e.g., beer, wine, diluted honey, juice), that wasps enter but cannot exit (e.g., 182, 183). The trapping methods that have been employed against *V. velutina* gynes have been criticized because they kill large numbers of non-target insects (178, 179, 184, 185). The use of species-specific attractants such as pheromones could alleviate this issue. Dong et al. (186) showed that *V. mandarinia* queen sex pheromones attract male hornets without bycatch, suggesting that tailored pheromonal trapping approaches could contribute to invasive hornet control.

There have been several attempts to find and destroy established nests of *V. velutina* during their summer active period, but before colonies produce reproductives. Because many nests are not detected (89, 185, 187), hornet populations generally continue to increase despite these control efforts (185, 188). Several tracking and identification methods have been employed to locate *V. velutina* colonies [e.g., visual tracking (183, 189); harmonic radar (190, 191); radiotelemetry (192); and video recordings coupled with artificial intelligence (193 and references therein)]. Looney et al. (33) employed radio-telemetry to track worker *V. mandarinia* hornets for distances up to 650 m and successfully locate their nests. All of these methods suffer from various limitations (194). Search times to locate individual colonies are lengthy (e.g., visual tracking: 19 days on average on Mallorca; harmonic radar: 2.5 days in Italy; radio telemetry: 2–3 hrs in the UK) (summarized in 190). Because of the small mass of individual hornets, signal-emitting tags that are sufficiently light-weight to be carried by a flying hornet have low signal ranges and short battery life (194). Costs are also a consideration: for example, the cost of harmonic radar equipment is ~100,000 Euros (190). Technological advances will undoubtedly bring down the mass of radio-transmitters [currently the minimum is ~150 mg (e.g., 195)] and increase their effectiveness over greater distances, thereby making radio-tracking of hornets more feasible in the future.

All methods for potentially controlling hornet populations, specifically *V. velutina* in Europe, were reviewed by Turchi and Derijard (169). On the mainland of Europe, the population of *V. velutina* is too well established to be eradicated, but a combination of trapping of gynes, removal of nests, and biological control may reduce the population size and its rate of spread (61). Other introduced populations of hornets with differing ecology may be more tractable to control. Perhaps the most promising techniques involve RNA interference and CRISPR genome editing (196, 197), methodologies that are still under development but may be as costly or labor intensive as other control strategies.

Eradication of an incipient population that experiences limited propagule pressure in a restricted geographic area, especially on an island with low probability of reinvasion, has a far greater chance of long-term success than does the elimination of an established population in a large region (198). In some situations, eradication early in an invasion may be more cost-effective than ongoing control efforts (198, 199). Actions taken early in the invasion process while infestations are still small and localized are critically important for a high probability of successful eradication (200–203). In several regions, populations of hornets detected soon after introduction have been successfully contained. For example, in England, great effort and considerable expense allowed cooperation between beekeepers, agricultural personnel, and scientists to locate and destroy nine *V. velutina* colonies between 2016–2019; no further nests were discovered during the following two years (139). However, the colonies detected in the UK represented multiple colonization events over several years (139). Immigration of gynes from mainland Europe is likely to continue and the exclusion of this species from the UK is almost certainly temporary. As a case in point, *V. velutina* was reported in 2021 in Ireland (204). In Mallorca, rapid response to early reports of *V. velutina* involved spring trapping of gynes, detection of foraging hornets at baits, and triangulation and destruction of nests, which resulted in the apparent eradication of *V. velutina* within four years of its initial detection (189). Ongoing efforts to trap gynes and locate and destroy nests of *V. velutina* on the Island of Guernsey, Channel Islands, may be reducing the population of the species there, but continuing immigration of gynes from the mainland prevents eradication (205). In North America, elimination of several nests of the recently invaded *V. mandarinia* may have helped to reduce the incipient exotic population (33). In all of these cases, extensive involvement by community residents and beekeepers, through the submission of their sightings, has been critical to these management successes.

Decisions related to nonnative insects have associated financial consequences because monitoring and control efforts are extremely costly activities (206). With respect to hornets, from 2004 (when *V. velutina* was first discovered in France) to 2016, destruction of nests in that country is estimated to have cost 23 million Euros, with costs projected to be 11.9 million Euros annually once the population has become fully established there (86). Annual costs in the UK and Italy are predicted to reach 9.0 million Euros and 8.6 million Euros, respectively (86). In Spain, where the species remains limited to the northern portion of the country, *V. velutina* has already become the eighth most-costly invasive species, with efforts designed to reduce the population and its rate of spread (207) estimated to cost $5.5 million USD per year (208). Furthermore, the goals of government agencies, scientists, and residents to address hornet invasions may not always align, thereby complicating the implementation of control strategies (207).

### 4.6 Climatic similarity

It has long been recognized that the establishment of an adventive species is more likely to occur if there is a strong similarity between the environments of the native range and site of introduction (209). The assemblages of species and their interactions will always differ between the regions, thereby complicating direct comparisons of a species in the two biotic communities. However, their abiotic environments, as defined by climatic variables (i.e., various aspects of temperatures and precipitation), can be readily analyzed and compared to gain insights into the likelihood of success of a species in non-native regions (210). The science of species distribution modeling (also referred to as climate envelope modelling, ecological niche modeling, etc.) has advanced rapidly since multiple regression analyses were applied to GIS-linked species distribution and digital environmental data (211). While different models (e.g., general linear models, maximum entropy models, etc.) differ in their algorithms, they all enable predictions of the probability of success of invasions in novel regions based on climatic variables in locations within the current range of a species (212, 213). They also can project changes in distributions under different climate-change scenarios (214). These models are based on climatic and species occurrence data (and species absence data in some models), and generally do not account for biotic factors such as habitat availability, competition with other species, and effects of predators and diseases, although additional variables can be added to some models (210, 212, 213; see 215 for an example related to hornets). The models vary in their assumptions and methodologies, and caution should be exercised when selecting models and interpreting the predictions they generate (211, 213). Modeling using relatively few geo-referenced occurrence localities for model development will suffer from a lack of data and may yield a poor prediction of the potential range of a species outside of its native range (210, 216, 217). Species distribution models can provide information that informs the design of surveys and control decisions for invading species (203).

The potential distributions of hornets beyond their endemic ranges have been modeled for only three species of hornets: *V. velutina*, *V. orientalis*, and *V. mandarinia*. There have been no attempts to model the potential distributions of the other two species that already have established introduced populations (e.g., *V. tropica*, *V. bicolor*) or species that are common in disturbed habitats and pose a high risk of successful invasion (e.g., *V. affinis*, *V. analis*, *V. simillima*). In some cases (e.g., *V. velutina*), different models agree on the potential distribution of a species, providing confidence in the predictions (78, 218). In other cases (e.g., *V. mandarinia*), the models agree broadly on the regions in the world that are susceptible to invasion but differ on details of the potential range at finer scales (219–223). Such discrepancies may reflect, in part, different sets of locality data used in model training and the various modeling approaches used. Researchers should be knowledgeable about the ecology of the invading hornet species and limitations of locality data in its endemic range prior to selecting their modeling approach(es) (210). Ensemble evaluations that combine results from two or more models (e.g., 78, 218) may improve predictive power. Details of the species distribution models that have been conducted on hornets appear in Sections 5.1, 5.2, and 6.

## 5 Successful invasions of *Vespa* species

Here we review the history and factors that have likely influenced the invasions by five *Vespa* species that have successfully established populations in nonnative localities (**Figure 1**). The common names we have used are those adopted by iNaturalist.org and the Entomological Society of America (224). The unfortunate designation of *V. velutina* as “the Asian hornet” created considerable confusion when another Asian hornet species, *V. mandarinia*, was discovered in North America. With all 22 species of *Vespa* occurring in Asia, “Asian hornet” is an uninformative misnomer.

### 5.1 *Vespa velutina*, the yellow-legged hornet

*Vespa velutina* is a common species of disturbed habitats, including urban areas (12), with a broad natural distribution. Its range extends from Pakistan eastward along the foothills of the Himalayas and across China to the eastern province of Shandong. Further to the south, it occurs in much of southern and eastern China, Taiwan Island, Southeast Asia, the Malay Peninsula, and from Sumatra eastward in the Indonesian archipelago to Timor and Sulawesi (46, 54, 225). Over that vast range, numerous geographic color forms have been recognized, formerly referred to as subspecies (226), that are associated with widely differing ecological conditions in no less than four biomes and 28 different terrestrial ecoregions (227) across its range. To date, only *V. velutina nigrothorax* is involved in the invasions of Europe and Korea+Japan.

*Vespa velutina* is the only hornet species that has established introduced populations that are presently considered to be invasive. In the EU and Japan, it meets the criteria of “invasive” by being a nonnative species that causes or has the potential to cause harm to the environment, biodiversity, economy, and human health (199, 228). South Korea has designated *V. velutina* as an “ecological disturbance organism” (13). Additionally, it has been recommended to be added to the World Organization for Animal Health’s list of diseases, infections, and infestations (229). That apparently one multiply mated gyne was able to establish a population in France that now inhabits most of mainland western Europe signals that this species is a formidable invader (62). Several reviews have summarized its history, status, invasion dynamics, and ecological impacts in Europe (17, 55, 96, 187, 188, 230, 231). In northeastern Asia, following its colonization of the Korean Peninsula in or before 2003 (12), it has dispersed over most of South Korea (232). It has also colonized Japan, beginning first on Tsushima Island in 2012 (57) followed by Kyushu Island in 2015 (233) and Iki Island in 2017 (68).

Villemant et al. (78, 185) modeled the potential range of *V. velutina* worldwide with multiple techniques. Because of *V. velutina*’s vast natural range, they used data for the geographic form of the hornet that invaded Europe and South Korea only in their models. The average climatic suitability values they predicted for locations in the invaded range in Europe were considerably smaller than but well within the range of values for suitability determined for sites within its endemic range in Asia. They stated that their climatic analyses would have predicted the successful invasion in southwestern France. However, their predictions of climatic suitability in France and other European countries were strengthened when climate data from localities in the invaded range in France were included in the model training data, suggestive of a niche-shift by the invading population (78) that is supported by a more recent study (234). The predicted distribution of *V. velutina* (78) has proven to be quite accurate in Spain, Portugal, and the island of Mallorca as the invasion has unfolded over time (17). Other climatic models for this species in the Iberian Peninsula (215) and for Europe and North Africa (235) yielded similar predictions to those of Villemant et al. (78). In South Korea and Japan, the regions that *V. velutina* has successfully colonized (59, 164, 233, 236) were also predicted to have suitable climates (78, 218). Species distribution modeling suggests that this hornet species will likely inhabit all of South Korea (232). Additional highly suitable climatic regions that are not yet inhabited by *V. velutina* have been identified in southeastern US, the Pacific Northwest of North America, southeastern South America, central Chile, southern Africa, southeastern and southwestern Australia, and New Zealand (78). Climatic suitability for *V. velutina* is projected to increase with climate change, resulting in increases to its potential distribution in many regions of the world (237).

As described previously, invading populations of *V. velutina* in both Europe and northeastern Asia exhibit impoverished genetic variability resulting from a small number of propagules that founded the populations, followed by significant bottleneck effects (58, 64, 68, 238, 239). Both populations trace their origins to eastern China (58, 64, 68). Despite low genetic diversity in the invading populations, *V. velutina* has proven to be a remarkably successful invader, with nest densities in parts of Europe of 5–6 nests/km^2^ (87, 188) and >12 nests/km^2^ in one urban environment (240). Peripheral populations in the Iberian Peninsula, northwestern Italy, and the United Kingdom have even further reduced genetic diversity compared to the population in France from which they are derived, indicating there have been no additional gynes introduced from Asia (125, 139), yet the species continues to expand its distribution in Europe. It is not yet clear which aspects of its biology have enabled its dramatic success there (241, 242), although a contributing factor may be its larger colonies and greater production of gynes compared to endemic *V. crabro* colonies (78). The invasion of South Korea has proceeded more slowly than that of France (12, 62, 90, 243). It has been suggested that this difference may reflect competition with sympatric hornet species (9, 12, 78, 244) and the relatively low position of *V. velutina* in the aggression hierarchy when they encounter other *Vespa* species in Korea and Japan (13). In contrast, *V. velutina* interacts and competes only with *V. crabro* in Europe (242). The slow initial range expansion in South Korea may also represent a time lag in population growth, as is commonly observed in recently founded populations, with several years of initially slow rates of population growth followed by significant increases in reproductive rate and dispersal (103, 105, 118). Interestingly, within seven years of colonizing South Korea near the port city of Busan, *V. velutina* had become more common there than the six endemic *Vespa* species (238).

In Europe, *V. velutina* has several ecological advantages over *V. crabro*: queens initiate ovarian development approximately one month earlier, colonies are more populous and produce more reproductives at the end of the season, and adult *V. velutina* prey extensively on honey bees that are both common and lack effective defenses (245–247). Behaviorally, *V. velutina* has the most extreme level of polyandry within the genus (**Table 2**) (140), and selection within its recently colonized range may favor increased mating frequency by gynes (155). Additionally, *V. velutina* gynes have greater immunocompetence against pathogenic bacteria than *V. crabro* gynes, a trait that may enhance their relative ecological success as competing invaders (248). Countering those factors, in laboratory tests, *V. crabro* workers exhibited greater antibacterial activity than *V. velutina* workers (242). At the population level, Carisio et al. (241) failed to detect any negative effects of *V. velutina* on *V. crabro* in Italy. In contrast, in South Korea, the increase in numbers of *V. velutina* has been correlated with a decline in abundance of *V. simillima*, a slightly smaller but ecologically similar species to *V. velutina* (13). In Japan, *V. velutina* numbers are negatively correlated with those of *V. simillima*, *V. analis*, and *V. mandarinia* (244). There is likely competition for prey among all these species, and especially for social insect prey between *V. velutina* and *V. mandarinia*. Moreover, *V. velutina* males mate interspecifically with gynes of *V. simillima*, thereby contributing to the reduction in abundance of *V. simillima* in South Korea following the invasion by *V. velutina* (249). A further factor is the predation by *V. mandarinia* on colonies of other *Vespa* species, particularly *V. analis* and *V. simillima*, and to a lesser extent, larger species (e.g., *V. crabro*, *V. tropica*) (71). *V. mandarinia* presumably also preys on *V. velutina* where the two species are sympatric.

*Vespa velutina* preys extensively on honey bees (especially *A. mellifera* L.), syrphid flies, social wasps, and other pollinators (17, 22, 182, 187, 241, 245, 246, 250–253). In some instances, intense predation by *V. velutina* workers foraging on insects at flowers can diminish overall pollination of plants (254). The presence of *V. velutina* hornets hovering at the entrance of a honey-bee hive suppresses foraging activity by *Apis mellifera* and contributes to decreased honey yields and greatly increased winter mortality (22, 255). The abundance of managed colonies of honey bees throughout most of Europe and the lack of effective defenses against the introduced hornet by *A. mellifera* have probably contributed to the success of this notorious bee predator.

Efforts to control invasive *V. velutina* in large geographic areas are unlikely to succeed (89, 180, 188). In contrast, attempts to eradicate *V. velutina* may be desirable and possible for adventive populations in regions where (i) the species has only just been detected and (ii) where propagule pressure is estimated to be low. Temporary eradication seems to have been accomplished in the UK and the Balearic Islands (139, 189), as described in section 4.5.

In summary, *V. velutina* has high invasion potential. It has large colonies that produce numerous reproductives and is abundant in regions with suitable climate (12). It preys extensively on honey bees, particularly relatively defenseless *Apis mellifera* (253), which are common over much of the world due to their management by beekeepers. Polyandry of gynes reduces the impacts of diploid-male production and increases the probability of establishment of introduced populations. Moreover, *V. velutina* populations thrive in human-modified environments (12), such as near many major ports in Asia and Western Europe, so its propagule pressure is likely high relative to other *Vespa* species. The most likely regions to become invaded, based on climate and volumes of commercial transoceanic shipping, are southeastern US and southeastern Australia.

### 5.2 *Vespa orientalis*, the oriental hornet

*Vespa orientalis* is a common hornet and the only species that inhabits arid environments (256). It occurs naturally from the eastern Mediterranean Basin through the Middle East to eastern India (257). In Europe, its endemic range includes the southern half of the Italian peninsula, Sicily, coastal Croatia, the Balkan Peninsula, Crete, Cyprus, and Turkey (258). *Vespa orientalis* has been accidentally transported outside of its endemic range to numerous localities on four continents (**Table 1**). In the past decade, *V. orientalis* seems to have successfully established populations in southern Spain and Gibraltar, possibly in two separate introductions in Andalucía (52) and València (69), and in northern Italy, in Tuscany (38, 40), Trieste (39), and Liguria (41). It was detected on the island of Sardinia for the first time in 2022 (41). Moreover, a population has expanded its range in west central Chile since it was first detected there in 2018 (35, 41). Other than these reports of range expansions, there are no published accounts related to the ecology and genetics of *V. orientalis* in these newly founded populations. Consequently, the geographic origins of these adventive populations are unknown, as is the extent to which some of the detections in Europe represent local jump-dispersal events versus independent introductions from more distant, potentially genetically differentiated, populations from further east in Europe, the Middle East, and East Asia.

Species distribution modeling confirms the high climatic suitability in the regions already colonized by *V. orientalis* in Chile and Spain (259). It also predicts additional regions in the world where the climate may be favorable, including the Gulf coast region of the United States and northeastern Mexico, central California, northwestern Argentina, southeastern Brazil, southeastern China, and parts of Australia (Figure S4.1 (b) of Werenkraut et al.; 259). Interestingly, several regions with historical individual records of this species where it failed to establish populations (e.g., French Guiana of France, Cozumel Island of Mexico, and Madagascar) were also predicted to be climatically unlikely to support populations of this species (259). The presence of *V. orientalis* in suitable climatic regions of Chile and Spain and its absence in unsuitable climates of French Guiana, Mexico, and Madagascar validate the utility of species distribution modeling for this species.

The large number of detections of *V. orientalis* outside its endemic range (**Table 1**) suggest that its propagule pressure may be high relative to that of other hornet species. Wintering of gynes in boxes and other containers (16) in addition to soil may be a contributing factor. Transportation of goods within Europe may have added to propagule pressure and range extensions detected recently into northern Italy, Sardinia, France, Romania, and elsewhere. Also, simultaneous transport of several gynes that are diapausing together (16) would increase propagule size and genetic variability, potentially helping founding populations to overcome initial genetic bottlenecks. This species is quite common and widespread in human-modified landscapes in many arid regions of the Middle East (76, 257). Although gynes are known historically to have stowed away in crates of citrus fruits shipped from the Middle East to western Europe (1, 67), the current contribution of this pathway of introduction to the range extension of *V. orientalis* is unclear.

The potentially high propagule pressure resulting from gynes diapausing in groups and in above-ground cavities, the abundance of this species in human-altered habitats, its demonstrated ability to colonize new regions (in Chile and Spain), and the many detections outside its natural range, collectively indicate that *V. orientalis* has high invasion potential. Mating frequency by gynes has not been documented; multiple mating per female would enhance its invasion potential by increasing the genetic diversity of colonists. Adventive populations of this species can be expected to arise in some of the dry regions with suitable climate identified by Werenkraut et al. (259).

### 5.3 *Vespa tropica*, the greater banded hornet

*Vespa tropica* has a broad natural distribution, from Afghanistan and Pakistan in the west to southeastern China, the Philippines, many islands of Indonesia, and New Guinea in the east (62). It was discovered recently, in 2016, on the Pacific Island of Guam (53) where it meets the criteria of being “invasive” (199). By the next year, the species was already sufficiently common that it was deemed to be established. Consequently, no controls were implemented other than the removal of nests that posed a risk to people. It has subsequently been recorded throughout the island (260). Its attacks on colonies of paper wasps (*Polistes stigma* (Fabricius) and *P. olivaceus* (De Geer)) as well as managed European honey-bee colonies on Guam (53) are likely causing environmental disruptions, but no details have been published. Genetic studies that could pinpoint the geographic origin(s) of the hornets on Guam or provide insights into the propagule pressure that resulted in the invasive population are lacking. It has long been speculated that islands, with their impoverished faunas, are particularly prone to invasions (261, 262), and Guam is no exception (263). Hopefully, future studies of the ecology, life history, and genetics of *V. tropica* on Guam will elucidate the history of the invasion and the main factors that have influenced its rapid success there.

The rapid takeover of Guam by *V. tropica* suggests that proactive climatic modeling could provide useful insight into other regions that are suitable for colonization by future invaders. The production of species distribution models that predict where this species might establish non-native populations would likely be complicated by the wide range of climatic conditions to which the 9–15 regional color forms (sometimes referred to as subspecies) of *V. tropica* are exposed across its expansive range (14, 65). Specification of the source population during model training may be necessary due to ecological differences between different geographic populations.

*Vespa tropica* is in the same phylogenetic clade as the giant hornet species, *V. mandarinia* and *V. soror* (141, 256), both of which usually mate with just one male (137, 138). If future research demonstrates that gynes of *V. tropica* also mate singly, that behavior would restrict its potential to establish exotic populations. On the other hand, tropical *V. tropica* colonies can be polygynous (74), thus a whole colony transported from a tropical locality has the potential to bring with it increased levels of founding genetic diversity. At present, many aspects of its biology are unknown, so it is difficult to estimate the worldwide invasion potential of *V. tropica*.

### 5.4 *Vespa bicolor*, the black shield hornet

*Vespa bicolor* is naturally distributed over much of southern China, Southeast Asia, and the foothills of the Himalaya Mountains (46, 226). Its first exotic detection, in 2003, was on Taiwan Island, only 5 km from Taichung Airport and 15 km from Taichung Harbor, both of which are major ports for importation of goods from continental Asia (48). More recent collections have been made in Sanyi, a community that imports large quantities of wood for its wood-carving industry. Since first being detected, the species has spread slowly in the hills and low mountains in the northern end of the island and is predicted to continue to spread southward in cool, hilly regions of Taiwan (47). Genetic analyses suggest that the population may have resulted from a single gyne introduced from southeastern China (47). On Taiwan, adult *V. bicolor* workers have been observed feeding on honeydew and floral nectar. To feed its larvae, adults were documented preying on honey bees (*A. mellifera*) by hovering in front of bee hives, as well as on solitary syrphid flies (48). Because of its modest population, *V. bicolor* is not considered to be a significant pest of honey bees on Taiwan at present (47).

In Spain, this species was first observed in 2013 in Málaga Province (27). It is suspected to have been accidentally transported from China (27), however no genetic analyses have yet confirmed that, nor have species distribution models to predict its potential range been developed. Initially the Spanish population was localized in a small region (27), but the range of the species has expanded considerably and by the end of 2022, observations had been reported over a linear distance of ~40 km (28). The size of its range suggests that the species is now locally established. 

The mating frequency of gynes of this species is unknown. However, *V. bicolor* is phylogenetically in the *bicolor* clade along with *V. simillima* and *V. velutina* (141), both of which typically exhibit multiple mating by gynes (**Table 2** and references therein). Data on mating frequencies among gynes are needed to better assess the likelihood of establishment of adventive populations of this species. As discussed previously, multiple mating increases the genetic diversity contained within individual gynes, and consequently reduces the genetic load imposed by diploid male production. If *V. bicolor* gynes do indeed mate with several males, the accompanying boost to genetic diversity would increase the likelihood of establishment of adventive populations of this species.

Species distribution models for *V. bicolor* are needed to determine the extent to which the Spanish population will expand its range in the Mediterranean region as well as what other regions in the world are susceptible to invasion.

### 5.5 *Vespa crabro*, the European hornet

The natural range of *V. crabro* spans temperate Eurasia, from Japan and far Eastern Russia to Europe, including southern Scandinavia, the UK, the Iberian Peninsula, and most of Italy (excluding Sardinia) (264). There have been two successful expansions of *V. crabro* outside of its endemic range. The first, in the US, began sometime around 1840 near port facilities in New York City (49). The species is presumed to have arrived there accidentally *via* stowaways in cargo shipped from Europe (49). It now occurs throughout much of eastern North America (31). In 2010, it was discovered on the island of Sardinia, Italy, possibly having arrived there *via* imports of wood from the mainland, and it now seems to be established on the northeastern end of the island (50, 265). Additionally, single live individuals of *V. crabro* were collected or photographed in Guatemala (30) and Mexico (31), and a dead individual was found on the west coast of Canada (29).

*Vespa crabro* is a generalist predator (242, 265) that has not been reported to cause significant environmental impacts anywhere within its natural or introduced ranges. It occasionally causes minor damage to branches it chews, presumably to obtain sap (9). It also preys on small numbers of honey bees but usually does not cause significant damage to their colonies (15, 265, 266). Recent studies have provided insights into differences in the ecology and behavior of *V. crabro* relative to the invasive *V. velutina* in northwestern Italy, in an attempt to better understand the very different abundances and impacts of these two species. One study detected no evidence of competition between *V. crabro* and *V. velutina* (241), perhaps because their niches and preferred habitats only partially overlap. Another found no substantial differences in behavioral traits (e.g., boldness, exploratory tendency, durations of foraging-related variables) (242). There has been no research to determine the provenances of the two exotic populations of *V. crabro* or genetic diversity within their introduced ranges.

## 6 *Vespa mandarinia*, the northern giant hornet, in North America

Wherever it occurs, *V. mandarinia* is regarded by the general populace as a fearsome predator with a potent and potentially lethal sting (267). Its endemic range extends from Hokkaido, Japan, and Primorsky Krai, Russia, south to Hong Kong, then westward across the southern edge of the Himalayas to northern India (268, 269). Within its endemic range in Asia, it is considered to be the most important predator of honey bees (18, 19). The endemic Asian hive bee, *Apis cerana*, has evolved several effective defenses against giant hornet attacks (270–275). In contrast, *A. mellifera* colonies are slaughtered unless hives are protected in some way (18, 19). Consequently, the detection in 2019 of several *V. mandarinia* workers and an active colony on Vancouver Island, Canada, followed soon after by five observations of worker hornets on the mainland in British Columbia and Washington state (29, 33), caused extreme concern due to the severe effects this species has on European honey bees as well as the risks giant hornets may pose to human health (267).

In the effort to eradicate the incipient *V. mandarinia* population, intensive survey efforts by government agencies combined with hundreds of community science reports over the next two years resulted in confirmation of 45 additional hornet sightings and specimens in British Columbia and Washington (35 in 2020; 10 in 2021), including the discovery and eradication of four colonies in Washington state (33). In 2022, no additional *V. mandarinia* individuals or nests were detected in that region of North America (33). DNA analyses indicated that the hornets that colonized North America had different origins, likely from Japan (Vancouver Island) and Korea (mainland sites) (163). Additionally, analysis of a male hornet found dead in WA in spring of 2021 suggested its provenance was China, which would represent another independent colonization event (276).

Based on the relatively small number of confirmed sightings and nests that have been detected in North America (33), the population of *V. mandarinia* in the Pacific Northwest has never been large. Additionally, the reduced numbers of sightings since 2020 suggest that the population is in decline. If that proves to be true, there will be no way to determine the exact cause(s) of this failed invasion. The four colonies that were eradicated in Washington state were small for this species (33) and consequently would have reared few reproductives relative to the numbers produced by larger colonies in warmer climates (21). Low genetic diversity resulting from a small number of colonizing gynes (i.e., small propagule size) that were likely singly mated (137), coupled with sl-CSD and the genetic load caused by rearing of diploid males, may be factors that are contributing to a population extinction vortex *(sensu* 158). Eradication of the colonies that were discovered also may have contributed to the apparent decline in this introduced population.

Four species distribution models that predict the potential range of *V. mandarinia* in the US and Canada have been created (219, 221–223). All of them agree relatively closely on the region suitable for *V. mandarinia* in the Pacific Northwest region where the species was discovered. In contrast, while the four studies also identified suitable climatic regions for this species in eastern North America, the exact regions where it would be most likely to survive differed substantially among models. The analysis of Alaniz et al. (219) was influenced by their assumption that *V. mandarinia* would prefer urban areas, which contradicts the evidence that it is predominantly an inhabitant of green spaces and forests in Japan and South Korea (277, 278). A similar preference for forested habitats has been observed for its sister species, *V. soror* (138, 279). However, both giant hornet species have infrequently been found nesting in human-built structures in urban or disturbed landscapes (279, 280).

A different approach to predict the potential range and associated risks of *V. mandarinia* in Washington and Oregon, US, incorporated estimates of dense forest biomass and plant hardiness zones (as proxies for suitable habitat and climate), proximity to ports (a proxy for probable sites of introduction), and density of apiaries (access to honey bees) (281). Range predictions based on plant hardiness zones are not likely to be as accurate as those resulting from climate modeling (282). However, adding plant hardiness zones to data analyzed with species distribution models may improve predictions (213).

Outside of the US and Canada, the projection for the potential range of *V. mandarinia* predicted specifically for Mexico (220) differs strikingly from the results of the other species distribution models (219, 221–223). The Mexican analysis included localities in Washington and British Columbia in the model training data (220) where the species has yet to become established. If the climate of that region is suboptimal for *V. mandarinia*, inclusion of those sites in the model may have affected the modeling results. There are also substantial discrepancies among the results of the models in the regions of South America, Africa, Europe, and Southeast Asia predicted to have suitable climatic conditions for *V. mandarinia* (220, 222, 223). The lack of agreement in the results of the species distribution models that have been conducted to date suggest that additional modeling is required to clarify the invasion potential of *V. mandarinia* outside of Asia.

Genetic analyses of individual specimens and of hornets collected from the colonies destroyed in Washington and BC will improve estimates of the numbers of founding gynes and confirm their provenances, as well as determine the number of times they had mated, diversity at the sex-determining locus, and the strength of the genetic bottleneck they experienced. Once these details have been established, these additional data may prove informative for predicting the outcomes of other incipient non-native populations of *Vespa* hornets.

## 7 Discussion

Numerous detections of nine *Vespa* species far from their endemic ranges (**Table 1**) have demonstrated that hornets have been extensively displaced to non-native regions through the activities of humans. Members of the genus *Vespa* share numerous life history traits that facilitate the establishment of new populations: mated gynes can be readily shipped long distances while they are in diapause; gynes self-disperse by flying considerable distances prior to nest initiation; their nests are constructed with readily available materials in non-specific locations; they have relatively high reproductive rates; most species are generalist predators with low habitat specificity; as eusocial insects, they exhibit considerable behavioral plasticity; they are large and well protected by their sting; and they are good competitors. Because they are conspicuous insects, their presence is often detected and documented, in part through records contributed by community scientists to open-access databases such as iNaturalist. However, we have sparse data on the numbers and the frequency of arrival of gynes (i.e., propagule pressure) *via* different pathways of introduction. While it is assumed that stowaway gynes on ships are the main propagules that get transported to exotic locations, they are infrequently intercepted. Moreover, inspections are rarely conducted systematically to enable estimates of propagule pressure (112). Many questions remain incompletely addressed. What numbers of hornets arrive in soil with nursery plants? Within packing materials? On ships versus in air cargo? From which countries? From what habitats? Attempts to understand future hornet introductions will be hampered without this information. In fact, compiling this review made clear how challenging it is for researchers to access governmental data about detections. As a counterexample, the intense survey and research efforts that have accompanied the *V. velutina* invasions in Europe and Asia demonstrate how much can be learned about an invader if stakeholders such as governmental officials, researchers, beekeepers, and naturalists cooperate to investigate hornet biology. Similarly, transparent actions taken swiftly by governments and community scientists following the detection of *V. mandarinia* in the Pacific Northwest have greatly contributed to our understanding of its incipient invasion into North America.

To date, with the exception of *V. velutina*, our understanding of the ecology of introduced species of hornets in their new exotic ranges is poor. Additionally, there is a tendency to consider all members of a species as roughly equivalent, a practice that has obvious pitfalls. Populations differ across the range of a species, sometimes strikingly. For example, the limited natural history information we have about *V. tropica* comes from Sumatra, Indonesia (74). Applying that knowledge to the invasion of Guam by *V. tropica* would be of little value if the hornets that colonized the island arrived from a location such as Hong Kong, Hanoi, or Chennai, and their ecology, behavior, and genetics in that source location differed greatly from those of the population on Guam. The only introduced *Vespa* species for which their likely origins have been determined are *V. velutina* (in both Europe and Korea+Japan), *V. bicolor* on Taiwan, and not-yet-established *V. mandarinia* in North America. This is a knowledge gap that needs to be filled if we hope to better understand and predict the outcomes of future hornet invasions. Species distribution modeling to predict regions where hornet species could likely establish new populations have been conducted only for *V. velutina*, *V. orientalis*, and *V. mandarinia*. Low numbers of geo-referenced locality records for some species will limit the effectiveness of climate modeling of potential ranges for uncommon hornet species.

We also lack details of hornet ecology and genetics early in the invasion process. That information would enable understanding of the dynamic processes at play and the evolution of specific traits important for invasion success (209). Scientists have begun to obtain that information for *V. velutina* (e.g., 58, 238) and *V. mandarinia* (e.g., 33, 163). The newly established populations of *V. orientalis* (in Spain and Chile) and *V. bicolor* (in Spain) offer valuable research opportunities. We know very little about the factors that affect the expansion of introduced populations after they have become established and the traits and conditions that favor dispersal (152). Diapause behavior may differ between species (e.g., group diapause in *V. orientalis* versus solitary diapause in most other species), and propagule pressure will be unique to each hornet introduction. Mating frequency of gynes is unknown for several species that have established non-native populations (e.g., *V. bicolor*, *V. orientalis*, *V. tropica*) and others likely to do so, yet it has a large effect on the genetic diversity represented within individual gynes and may be a critical factor in the success or failure of invading populations that develop from very few immigrant gynes. All of these biological characteristics influence the potential threat of hornet invasions. One thing is certain: the constant global movement of goods will facilitate future invasions by hornets, particularly common species with polyandrous gynes (9, 58) and broad niches (209), traits that permit hornets and their colonies to thrive in human-altered environments.

## Author contributions

GO: Conceptualization, Original draft preparation. BT and HM: Contributions to original text. All authors: Editing, Review, Figure construction. All authors contributed to the article and approved the submitted version.
