# Corrigendum: Invasion potential of hornets (Hymenoptera: Vespidae: *Vespa* spp.)

**DOI:** 10.3389/finsc.2023.1253176

**Published:** 2023-09-28

**Authors:** Gard W. Otis, Benjamin A. Taylor, Heather R. Mattila

**Affiliations:** ^1^ School of Environmental Sciences, University of Guelph, Guelph, ON, Canada; ^2^ Institute of Bee Health, Vetsuisse Faculty, University of Bern and Agroscope, Bern, Switzerland; ^3^ Department of Entomology, Purdue University, West Lafayette, IN, United States; ^4^ Department of Biological Sciences, Wellesley College, Wellesley, MA, United States

**Keywords:** Asian hornet, extinction vortex, giant hornet, invasion potential, invasive species, propagule pressure, *Vespa*

In the published article, there were three errors. *Vespa tropica* has been split into two species, *V. tropica* and *V. ducalis*, and the authors failed to realize this. *Vespa ducalis*, as currently recognized, occurs in temperate regions such as Japan and Korea, whereas *V. tropica* occurs in tropical and subtropical regions. Therefore, our contrasts of tropical and temperate *V. tropica* populations were unfounded.

A correction has been made to **5 Successful Invasions of *Vespa* spp.**, *5.3 Vespa tropica, the greater banded hornet*, Paragraph 1. This sentence previously stated: “*Vespa tropica* has a broad natural distribution, from Afghanistan and Japan in the north to the tropical islands of Indonesia, the Philippines, and New Guinea in the south (46).”.

The corrected sentence appears below:

“*Vespa tropica* has a broad natural distribution, from Afghanistan and Pakistan in the west to southeastern China, the Philippines, many islands of Indonesia, and New Guinea in the east (62).”

A correction has been made to **5 Successful Invasions of *Vespa* spp.**, *5.3 Vespa tropica, the greater banded hornet*, Paragraph 2. This sentence previously stated: “Specification of the source population during model training may be necessary due to ecological differences between temperate and tropical populations.”.

The corrected sentence appears below:

“Specification of the source population during model training may be necessary due to ecological differences between different geographic populations.”

A correction has been made to **7 Discussion**, Paragraph 2. This section previously stated: “For example, the natural history information we have about *V. tropica* comes predominantly from studies in Japan. However, applying that knowledge to the invasion by this species in Guam would be of little value if the hornets that colonized the island arrived from a tropical locality such as Manila, Bangkok, or Chennai. For instance, mature *V. tropica* colonies in Japan are monogynous, have combs with a few hundred cells, and rear only a few dozen new gynes (2). In contrast, colonies in tropical Sumatra may be polygynous, construct more than 5000 cells, and produce several hundred gynes (74).”.

The corrected section appears below:

“For example, the limited natural history information we have about *V. tropica* comes from Sumatra, Indonesia (74). Applying that knowledge to the invasion of Guam by *V. tropica* would be of little value if the hornets that colonized the island arrived from a location such as Hong Kong, Hanoi, or Chennai, and their ecology, behavior, and genetics in that source location differed greatly from those of the population on Guam.”

The authors apologize for these errors and state that they do not change the scientific conclusions of the article in any way. The original article has been updated.

